# Quiet quitting: A significant risk for global healthcare

**DOI:** 10.7189/jogh.13.03014

**Published:** 2023-03-31

**Authors:** Yasemin Boy, Mahmut Sürmeli

**Affiliations:** 1 Department of Nursing, Faculty of Health Sciences, Tokat Gaziosmanpasa University, Tokat, Turkey; 2 Department of Physiotherapy and Rehabilitation, Faculty of Health Sciences, Tokat Gaziosmanpasa University, Tokat, Turkey

## QUIET QUITTING: A SIGNIFICANT RISK FOR GLOBAL HEALTHCARE

As the world faced the COVID-19 pandemic, most of us did not expect it to so profoundly affect all aspects of human life. Sudden decisions on social isolation rules and lockdowns significantly disrupted labour in all sectors and industries, beyond the scope of the global health crisis. Consequently, companies and employees rapidly adopted remote working models. This led many employees to recognize the benefits of remote work, such as flexibility, comfort, and work-life balance [1]. However, job and wage inequities, increased workload, and role conflicts have also emerged frequently in the business world. This altered and disrupted employees' work attitudes, habits, and behaviours, resulting in burnout, turnover intention, and disengagement. Consequently, many people in both scenarios have started resigning from their existing positions, including tens of millions of individuals in the US. This movement, rapidly termed “The Great Resignation”, has occupied media headlines and spread rapidly beyond the US, including, but not limited to, Europe and Asia – albeit to a lesser extent. Indeed, European authorities claimed that employment participation rates were above pre-pandemic levels. A similar, albeit smaller effect was observed in Asia [2].

**Figure 1:**
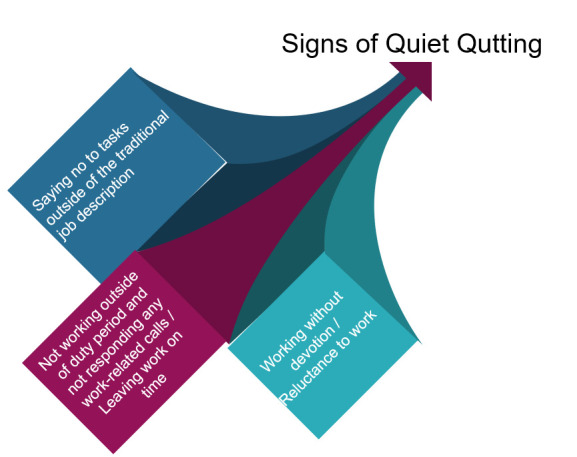
The basic summary of the actions of quiet quitters. From the authors’ own collection.

Even though that movement is decreasing, people are still in need of a better work-life balance. This has led to another tendency called “quiet quitting” or “silent resignation” in the business world. It does not refer to quitting a job, but rather indicates an adopted work behaviour [3]. The employees “only” perform the assigned tasks within their job description without extra effort and working devotedly. They do not intend to exceed their baseline obligations; they choose to perform all their tasks during working hours, rejecting the mentality of being available for more work after hours. In other words, they adopt the motto “working to live” instead of “living to work". It allows employees to set boundaries between work and personal life. They work with the understanding of “leaving work at work” to save energy for their social lives (**Figure 1**). Not all employees have the option to leave their job, which will most likely lead individuals who return to their positions after resigning or are unable to resign to adopt this attitude [4]. 

While it has been adopted in most sectors, the remote working model was not applied to health workers (HWs). The surge of cases and shortages of medical staff and equipment led to insufficient response to the urgent crisis in health care demands. Consequently, HWs have had to face many challenges since the beginning of the pandemic crisis [5]. Therefore, it is crucial to address the tremendous difficulties faced by HWs and their concerns for their health.

Although the recruitment of HWs increased in most countries, the massive wave of resignations is expected to inevitably hit the health sector. Female workers with children, younger, primary care, and frontline HWs constitute the largest group willing to turn over. Additionally, HWs who are not willing to leave the health sector may pursue alternative careers in the same or different professions with reduced work hours and workload [6,7]. It is easy to understand the reasons behind the rise in HWs’ work behaviour changes. Significant risk of infection, adverse working conditions, economic recession, inflation, disparities in workload and payment, toxic organizational culture, physical and verbal violence, anxiety, depression, burnout, and the consequent disruption of work-life balance seem to have caused the drastic change in HWs’ attitudes towards maintaining a work-life balance and increasing the quality of life [1,3]. Challenges regarding the management of daily living, which led to the disruption of a work-life balance, have contributed to decreased work engagement and increased stress. Being away from social life and being afraid of transmitting the infection to family members also made HWs think about work conditions and job satisfaction [7].

Despite efforts to increase HWs’ motivation, the inability to manage these issues highlighted that the toxic organizational culture could be a significant barrier to improvement efforts. Uncertainty, a lack of well-structured action plans, communication problems, inequalities in workload, income, protective equipment (whose distribution was based on profession and seniority), lack of appreciation by colleagues or patients, resignation and annual leave restrictions, as well as associated factors leading to HWs feeling as victims of injustice all contributed to the changes in work attitudes and behaviours [2,7]. Beyond that, increased exposure to physical violence is perhaps the most crucial factor that should not be ignored. In fragile countries with toxic organizational structures, the absence of strict laws has fuelled the rates of violence and put health workers in an unfavourable position [8]. In fact, the idea that they are more likely to be punished in case of a complaint despite being subjected to violence or that the legal sanctions against the perpetrator of the violent act are not going to not work was adopted by some HWs and led to an increase in psychological problems. They began to question their next steps, reconsider their lives, and strive to achieve a work-life balance, particularly due to concerns about life expectancy and the place of employment.

**Figure:**
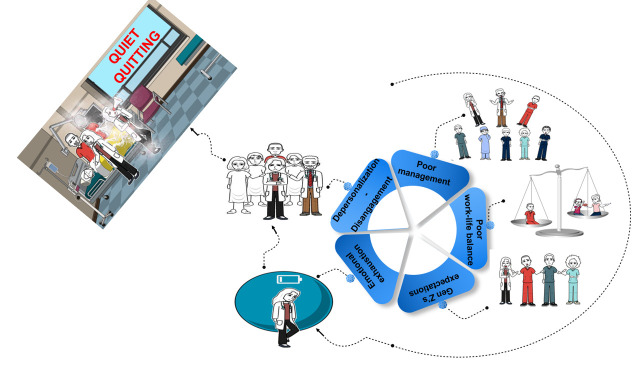
Photo: The main situations that led to the development of quiet quitting. Differences in the expectations of the Z generation, poor management, and poor work-life balance lead to emotional exhaustion over time; emotional exhaustion leads to disengagement and depersonalization over time; disengagement and depersonalization lead to quiet quitting over time. From the authors’ own collection.

The following question may come to mind: “Does quiet quitting pose a significant threat even if there is no reduction in the number of employees?”. Considering the increasing rate of young people in the workforce and the greater adoption of this new trend globally, the answer would be “yes”.

The resignation movement is still going on and will not be stopped. Over the next several years, the deficit of HWs will reach millions. In quiet quitting, employees act only within their job descriptions, without passion and work commitment. When considering the increasing ratio of young employees, we should critically analyse the future of health care quality. Cooperation between patients and health care providers and a well-structured environment are strong determinants of health care quality. Therefore, improving organizational culture is essential for appropriate attitudes, behaviours, enjoyment, and engagement for organizational members [9]. Some personal characteristics such as compassion, devotion, empathy, therapeutic touch, a gentle attitude, and excellent communication are necessary but not listed in the job description for HWs. By considering the rise in the workforce rate of Gen Z members in countries within the Organization for Economic Co-operation and Development (OECD), it is necessary to foresee that the following generations will shape the changes in the attitudes and behaviours of HWs and that they will be more dependent on the work-life balance. While they are not opposed to corporate institutionalism, they favour flexibility, being appreciated, autonomy, and work-life balance, and are more eager to work remotely and less prone to working devotedly [10]. By considering generational differences, it becomes clear that we need to improve health care quality by implementing organizational improvements to promote business involvement.

To sum up, HWs have faced the risk of infection, adverse working conditions, physical and verbal violence, disparities in workload and payment, limitations to attend social activities, and disruption in work-life balance for a long time. The inadequacy of attempts to solve the issues caused them to change their work attitudes and behaviours. Current trends emphasize the importance of understanding the reasons behind the employees’ resignations and how they can be prevented on time. The new trend of “quiet quitting” has been adopted in many countries, especially among young employees, and could adversely affect health care quality by triggering a toxic organizational culture.

The outbreak sparked radical changes in all sectors of human activity, including health care. We have learned the importance of protecting and improving health besides patient-specific clinical aspects; we need well-structured organizational culture, a strong and well-founded economy, well-equipped health centres and health workers, proper policy practices, and many more. Health systems are just one area where fundamental changes should be made before it is too late. HWs are essential to the functioning of health systems; expanding health care coverage and attaining the right to the highest possible level of health are based on the availability, accessibility, acceptance, and quality of health care. Policymakers must take the necessary steps to improve health care quality by considering gender, family, profession, and age group differences in line with technological, scientific, and social developments. Additionally, we need to remember the factors that reduce organizational commitment, job satisfaction, productivity, and motivation. Acting in the international cooperation framework will contribute to greater harmony between societies.

## References

[1] Shukla V Pandiya B Gupta S Prashar S The Great Resignation: An Empirical Study on Employee Mass Resignation and its Associated Factor. Research Square. 2022. 10.21203/rs.3.rs-1690874/v1

[2] Braje IN. Can Toxic Organizational Culture Really Cause the Great Resignation: In Search of Answers. 3^rd^ International Conference on Innovative Approaches in Social Sciences, Economics and Business Management. 2022:1-94.

[3] Formica S Sfodera F The Great Resignation and Quiet Quitting paradigm shifts: An overview of current situation and future research directions. J Hosp Mark Manag. 2022;31:899-907. 10.1080/19368623.2022.2136601

[4] Yikilmaz İ. Quiet Quitting: A Conceptual Investigation. In: Gürçay G, Manafidizaji A, editors. Anadolu 10^th^ International Conference on Social Sciences Proceedings Book; 2022; Diyarbakir, Turkey. Academy Conferences Publishing House:581-591.

[5] Poon YR Lin YP Griffiths P Yong KK Seah B Liaw SY A global overview of healthcare workers’ turnover intention amid COVID-19 pandemic: a systematic review with future directions. Hum Resour Health. 2022;20:70. 10.1186/s12960-022-00764-7 36153534 PMC9509627

[6] Frogner BK Dill JS Tracking Turnover Among Health Care Workers During the COVID-19 Pandemic. JAMA Health Forum. 2022;3:e220371. 10.1001/jamahealthforum.2022.0371 35977315 PMC8994131

[7] Ayaslıer AA Albayrak B Çelik E Özdemir Ö Özgür Ö Kırımlı E Burnout in primary healthcare physicians and nurses in Turkey during COVID-19 pandemic. Prim Health Care Res Dev. 2023;24:e4. 10.1017/S146342362200069X 36617850 PMC9884527

[8] Özdamar Ünal G İşcan G Ünal O The occurrence and consequences of violence against healthcare workers in Turkey: before and during the COVID-19 pandemic. Fam Pract. 2022;39:1001-8. 10.1093/fampra/cmac024 35395085 PMC9383775

[9] Mosadeghrad AM Factors influencing healthcare service quality. Int J Health Policy Manag. 2014;3:77-89. 10.15171/ijhpm.2014.65 25114946 PMC4122083

[10] World Economic Forum. Annual Report 2021-2022. Available: https://www.weforum.org/reports/annual-report-2021-2022/ Accessed: 15 January 2023.

